# Shared recognition of citrullinated tenascin-C peptides by T and B cells in rheumatoid arthritis

**DOI:** 10.1172/jci.insight.145217

**Published:** 2021-03-08

**Authors:** Jing Song, Anja Schwenzer, Alicia Wong, Sara Turcinov, Cliff Rims, Lorena Rodriguez Martinez, David Arribas-Layton, Christina Gerstner, Virginia S. Muir, Kim S. Midwood, Vivianne Malmström, Eddie A. James, Jane H. Buckner

**Affiliations:** 1 Center for Translational Immunology, Benaroya Research Institute at Virginia Mason, Seattle, Washington, USA.; 2 Kennedy Institute of Rheumatology, Nuffield Department of Orthopaedics, Rheumatology and Musculoskeletal Sciences, University of Oxford, Oxford, United Kingdom.; 3 Division of Rheumatology, Department of Medicine, Center for Molecular Medicine, Karolinska Institutet, Karolinska University Hospital Solna, Stockholm, Sweden.; 4 Center for Systems Immunology, Benaroya Research Institute at Virginia Mason, Seattle, Washington, USA.

**Keywords:** Autoimmunity, Immunology, Beta cells, Rheumatology, T cells

## Abstract

Tenascin-C (TNC), an extracellular matrix protein that has proinflammatory properties, is a recently described antibody target in rheumatoid arthritis (RA). In this study, we utilized a systematic discovery process and identified 5 potentially novel citrullinated TNC (cit-TNC) T cell epitopes. CD4^+^ T cells specific for these epitopes were elevated in the peripheral blood of subjects with RA and showed signs of activation. Cit-TNC–specific T cells were also present among synovial fluid T cells and secreted IFN-γ. Two of these cit-TNC T cell epitopes were also recognized by antibodies within the serum and synovial fluid of individuals with RA. Detectable serum levels of cit-TNC–reactive antibodies were prevalent among subjects with RA and positively associated with cyclic citrullinated peptide (CCP) reactivity and the HLA shared epitope. Furthermore, cit-TNC–reactive antibodies were correlated with rheumatoid factor and elevated in subjects with a history of smoking. This work confirms cit-TNC as an autoantigen that is targeted by autoreactive CD4^+^ T cells and autoantibodies in patients with RA. Furthermore, our findings raise the possibility that coinciding epitopes recognized by both CD4^+^ T cells and B cells have the potential to amplify autoimmunity and promote the development and progression of RA.

## Graphical abstract

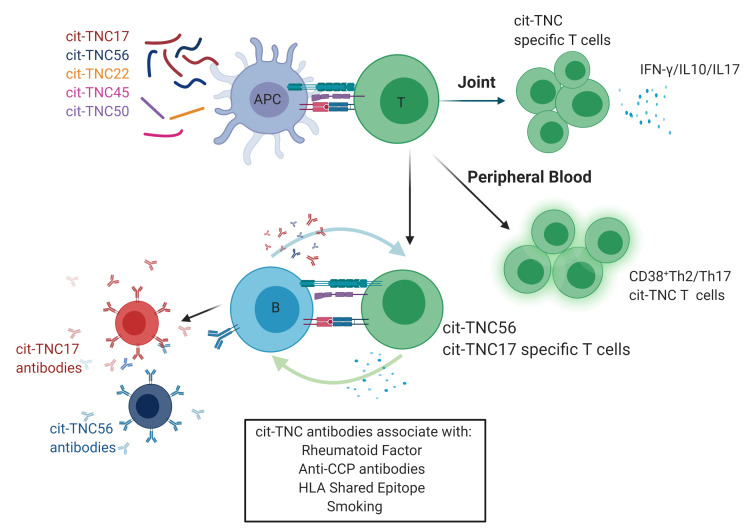

## Introduction

Rheumatoid arthritis (RA) is a chronic debilitating disease with a prevalence between 0.3% and 1% (1). It is a systemic, inflammatory autoimmune disease, with joints as the primary site of inflammation, and its progression leads to significant morbidity and increased mortality in affected individuals. Anti-citrullinated protein antibodies (ACPA) are hallmarks of RA and are predictive of developing disease, thereby defining an at-risk population (2–4). RA has a strong genetic association with the shared epitope (SE), a group of HLA-DRB1 alleles with a common motif in residues 70–74 of their peptide binding groove (5). The high level of somatic mutation in ACPA suggests an important role for ongoing T cell help in the disease (5–7). Indeed, a variety of CD4^+^ T cell specificities have been implicated in disease pathogenesis, including multiple citrullinated peptides from self-proteins that have been shown to be recognized not only by peripheral blood T cells, but also by T cells from synovial fluid (8–11). In spite of this knowledge, the immunologic factors that initiate and perpetuate RA are not completely understood. Perhaps due to discovery bias, there is broad overlap between antigens that are known ACPA targets and known sources of T cell epitopes. However, this overlap, coupled with the observation of compartmentalized T and B cell structures, could indicate that interactions between antigen-specific T cells and B cells play an important role in the etiology of RA (12).

Among the diverse array of citrullinated antigens that have been reported, published work implicates extracellular matrix proteins (e.g., fibrinogen, cartilage intermediate layer protein, fibronectin, type II collagen, and aggrecan) as an important class of citrullinated antigens, implying an important role in disease etiology (9, 10, 13–15). There is growing evidence that the extracellular matrix protein, tenascin-C (TNC) is an important citrullinated antigen in RA. TNC protein is largely absent in healthy tissues, but its expression is upregulated in response to inflammation in multiple autoimmune disease settings (16–19). In patients with RA, TNC protein levels are elevated in serum, as well as in the synovium of knee joints, correlating with disease activity (17). In addition to its known effects as a TLR4 agonist (20), the C-terminal fibrinogen-like domain of TNC has been shown to be citrullinated within synovial fluid (21), and immunodominant peptides within this domain are recognized by serum ACPA from subjects with RA (22). Notably, the presence of citrullinated TNC (cit-TNC) autoantibodies in an early synovitis cohort was associated with development of RA (23). These studies establish TNC as a relevant autoantigen in RA and highlight the potential application of measuring TNC specific immunity as a biomarker of disease activity and a means of understanding the loss of tolerance. However, it remains unclear which domains of this complex protein are immunogenic and whether antibody and T cell responses are most prevalent in a particular subset of subjects with RA.

Therefore, in this study, we sought to examine CD4^+^ T cell responses to cit-TNC by screening the entire length of the TNC protein to identify potentially novel T cell epitopes recognized in the context of the prevalent RA susceptible HLA-DRB1*04:01 allele. We identified 5 immunogenic cit-TNC peptides that elicited T cell responses in the peripheral blood and synovial fluid of RA subjects. Additionally, 2 of these immunogenic cit-TNC peptides were also antibody targets that were detectable within peripheral blood and synovial fluid samples from individuals with RA. This work demonstrates the importance of cit-TNC as an autoantigen in RA and establishes a link between epitopes that drive CD4^+^ T cell responses restricted by a high-risk SE allele (HLA-DRB1*04:01) and antibody epitopes that are recognized in APCA^+^ RA.

## Results

### Cit-TNC peptides bind HLA-DRB1*04:01 and are immunogenic.

TNC is a large protein (2110 amino acids in length) composed of an N-terminal assembly domain, 14.5 epidermal growth factor–like repeats (EGF-L), 8 constant fibronectin type III–like repeats (FNIII), and an additional 9 alternatively spliced FNIII elements, and a C-terminal fibrinogen-like globe (FBG) (16). Using a previously described algorithm (9, 24), we scanned the entire length of the TNC monomer and identified 64 arg-containing peptides with motifs that would predict to permit binding to HLA-DRB1*04:01 in either their noncitrullinated native form, citrullinated form, or both. Based on these predictions, we synthesized the citrullinated versions of these peptides and tested their ability to bind HLA-DRB1*04:01 and to elicit CD4^+^ T cell expansion in vitro. Of the peptides synthesized, 8 cit-TNC peptides bound to HLA-DRB1*04:01 with moderate to high affinity (Table 1). Among these, cit-TNC17, cit-TNC22, cit-TNC45, cit-TNC50, and cit-TNC56 were determined to be the most immunogenic, based on their ability to drive epitope-specific T cell expansion, as measured by HLA class II tetramer (Tmr) staining of peripheral blood mononuclear cells (PBMC) from HLA-DRB1*04:01^+^ subjects following 2 weeks of in vitro expansion (Table 1 and Supplemental Figure 1; supplemental material available online with this article; https://doi.org/10.1172/jci.insight.145217DS1).

**Figure 1:**
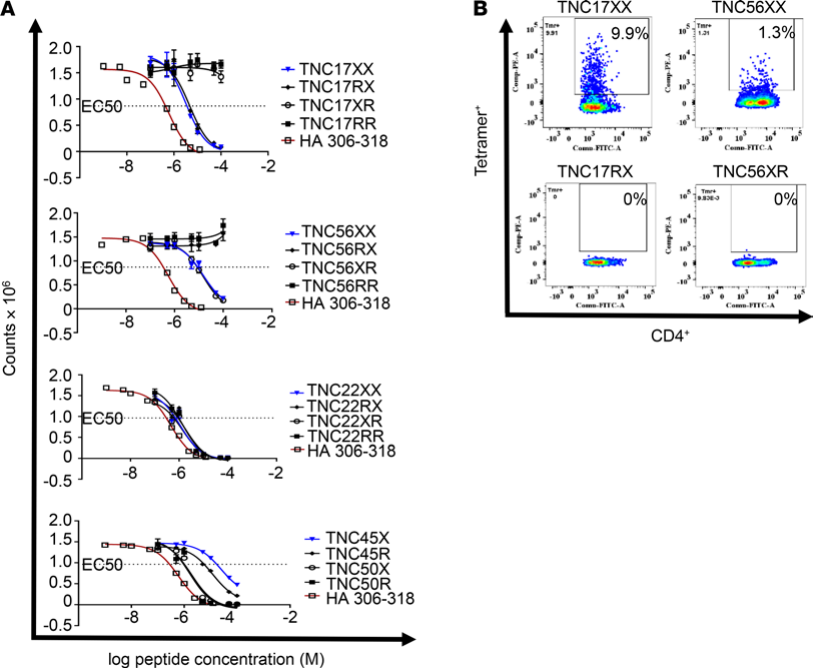
Citrulline residues are required in cit-TNC17 and cit-TNC56 peptides for HLA-DRB1*0401 binding and immunogenicity. (**A**) The binding of cit-TNC and their counterpart arginine peptides (arg-TNC) to DRB1*04:01 was tested using a peptide competition assay; each peptide was tested in triplicate. Europium-conjugated streptavidin was used to label residual biotinylated peptide bound to the HLA-DR protein. Binding curves were fitted by nonlinear regression with a sigmoidal dose response curve model, and EC_50_ values were calculated as the peptide concentration needed to displace 50% of the reference peptide. The HA306–318 peptide was used as a positive control. The EC_50_ cutoff for measurable binding was 50 μM. Results indicated that citrullination at 1 specific residue was required for cit-TNC17 and cit-TNC56 binding. X stands for 1 citrullinated amino acid in cit-TNC; R stands for 1 arginine amino acid in arg-TNC. (**B**) Citrullination of a distinct T cell contact residue was associated with cit-TNC17 and cit-TNC56 immunogenicity. Representative plots showing representative CD4^+^ T cell response to TNC peptide citrullinated at either both residues (TNC17XX and TNC56XX) or only 1 residue (TNC17RX and TNC56XR); *n* = 14 RA subjects tested for all peptides.

To assess whether citrullination is required for the HLA binding and immunogenicity of these peptides, the corresponding unmodified arginine-containing peptides (arg-TNC) were synthesized. Among the arg-TNC peptides synthesized, arg-TNC22, arg-TNC45, and arg-TNC50 bound to HLA-DRB1*04:01, whereas arg-TNC17 and arg-TNC56 did not (Figure 1A and Supplemental Table 1). This suggests that cit-TNC17 and cit-TNC56 are citrullinated at sites that are critical for binding. Indeed, the cit-TNC17 and cit-TNC56 peptides each contain a citrulline residue that is predicted to bind in a pocket 4 of HLA-DRB1*04:01. To determine whether any of the citrullinated sites are critical for TCR recognition, we assessed the immunogenicity of the arg-TNC22, arg-TNC45, and arg-TNC50, observing that each of these noncitrullinated versions had the ability to elicit T cell responses in vitro (Supplemental Table 1). We also investigated partially citrullinated versions of cit-TNC17 and cit-TNC56, showing that each contains a predicted TCR contact that is associated with increased immunogenicity (Figure 1B and Table 2). These findings indicate that TNC has the ability to promote T cell responses in both its citrullinated and noncitrullinated forms, but that some epitopes are uniquely immunogenic when citrullinated.

### The frequency of cit-TNC–specific CD4^+^ memory T cells is increased in RA subjects.

**Figure 2:**
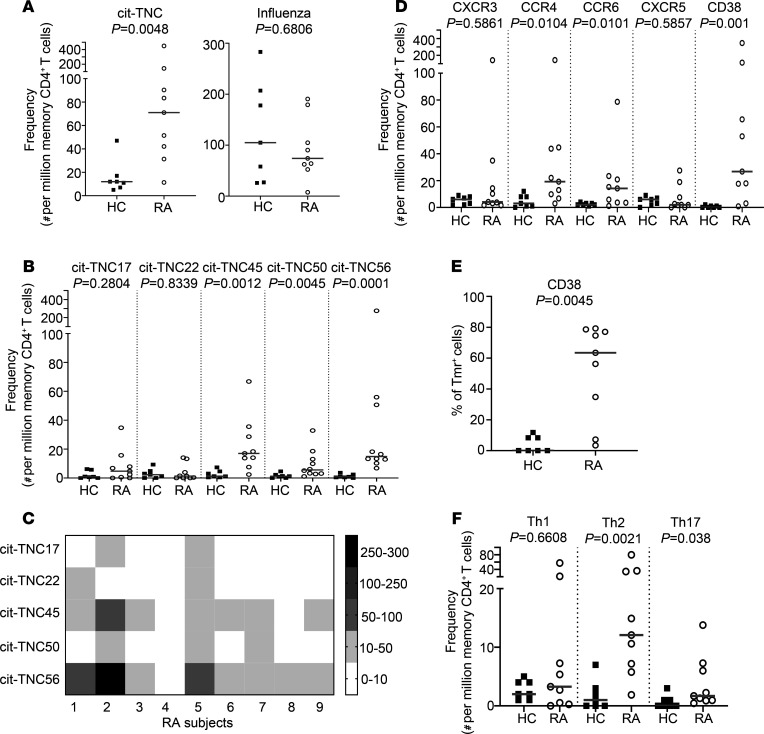
T cells that recognize tenascin-C epitopes are more frequent and have a distinct phenotype in patients with RA. The frequency and phenotype of tetramer^+^ cit-TNC–specific T cells was determined ex vivo using a multiplex HLA class II tetramer approach including cell surface marker antibodies to define the phenotype. Frequencies are expressed as the number of antigen-specific cells per million CD4^+^ memory T cells. Each symbol represents an individual subject (*n* = 7 for HC, *n* = 9 for RA), and the horizontal bar shows the median. (**A**) The frequencies of the combined cit-TNC–specific CD4 memory T cells were elevated in patients with RA compared with HC subjects, but the frequencies of influenza-specific CD4^+^ memory T cells were similar between RA and HC subjects. (**B**) The increased frequency of cit-TNC–specific CD4^+^ memory T cells appeared to be mainly due to cit-TNC45, cit-TNC50, and cit-TNC56. (**C**) The heatmap shows the cit-TNC T cell frequency for each epitope in each subject. Each row stands for a single epitope, and each column stands for 1 RA subject; intensity based on the number of cit-TNC T cells per million CD4^+^ memory T cells. (**D**) The frequencies of CCR4^+^, CCR6^+^, and CD38^+^ cit-TNC–specific CD4^+^ memory T cells were elevated in patients with RA compared with HC subjects. (**E**) The percent of CD38^+^ cells among total cit-TNC–specific memory CD4^+^ T is increased in patients with RA compared with HC subjects. (**F**) The lineage of cit-TNC–specific CD4^+^ memory T cells in RA subjects was predominantly Th2 (CCR4^+^CCR6^–^CXCR3^–^) and Th17 (CCR4^+^CCR6^+^CXCR3^–^), but not Th1 (CXCR3^+^CCR4^–^CCR6^–^). *P* values were calculated using an unpaired nonparametric Mann-Whitney *U* test.

We next asked whether cit-TNC–specific T cells are present in individuals with RA (*n* = 9) and healthy control (HC) subjects (*n* = 7) (Supplemental Table 2; T cell cohort). We directly assessed the frequency and surface phenotype of the cit-TNC–specific T cells using a multiplex HLA class II Tmr staining approach that allows ex vivo enrichment and detection of multiple Tmr specificities in a single peripheral blood sample and costaining with cell surface marker antibodies (10). The flow cytometry panel included the 5 immunogenic cit-TNC specificities — cit-TNC17, cit-TNC22, cit-TNC45, cit-TNC50, and cit-TNC56 — plus an influenza peptide (MP 97-116) as a positive control. Combining all cit-TNC specificities, there was a significant increase in the frequency of memory cit-TNC–specific T cells in patients with RA compared with HC subjects (Figure 2A). In contrast, the frequency of influenza-specific memory T cells did not differ between RA and HC subjects. Notably, cit-TNC45, cit-TNC50, and cit-TNC56 contributed the most to the increased frequency of cit-TNC–specific T cells, with a trend toward higher cit-TNC17–specific T cells that did not reach statistical significance (Figure 2, B and C, and Supplemental Table 3). In contrast, the frequency of influenza-specific memory T cells did not differ between RA and HC subjects. Notably, the frequencies of cit-TNC45–, cit-TNC50–, and cit-TNC56–specific T cells were all significantly increased, while the other specificities were only detected in a few of the RA patient samples (Figure 2, B and C, and Supplemental Table 3). As can be seen in Figure 2C, each patient sample contained several cit-TNC T cell specificities but with variable frequencies.

### Cit-TNC–specific T cells are recently activated and have predominantly Th2 and Th17 phenotypes.

We utilized the cell surface phenotype of cit-TNC–specific T cells in peripheral blood to draw inferences about T cell lineage, observing key differences between RA subjects and HC subjects. The cell surface markers included CD45RA as a marker of naive T cells, CD38 as a marker of recent activation, and CXCR3, CCR4, CXCR5, and CCR6 to define Th1, Th2, Tfh, and Th17 Th subsets, respectively (25–27). Most notably, the frequency and percentage of CD38^+^cit-TNC–Tmr^+^CD4^+^ memory T cells were increased in RA compared with HC subjects (Figure 2, D and E). On memory cells, this marker has been shown to indicate in vivo activation due to antigen exposure (28, 29). Therefore, the presence of CD38 suggests that a substantial proportion of cit-TNC–specific T cells in RA subjects were recently activated. In addition, there was a significant increase in the frequency of both CCR4^+^ and CCR6^+^ cit-TNC–specific T cells in RA subjects compared with HC subjects (Figure 2D). Further gating of these Tmr^+^ cells indicated that this represented a significant increase in the Th2 (CCR4^+^CCR6^–^CXCR3^–^) and Th17 (CCR4^+^CCR6^+^CXCR3^–^) population within the RA cohort (Figure 2F). Therefore, our results suggest that cit-TNC–specific T cells are more frequent in subjects with RA than in controls and exhibit an activated Th2/Th17 phenotype in peripheral blood.

### Synovial fluid mononuclear cells secrete cytokines in response to cit-TNC peptides.

**Figure 3:**
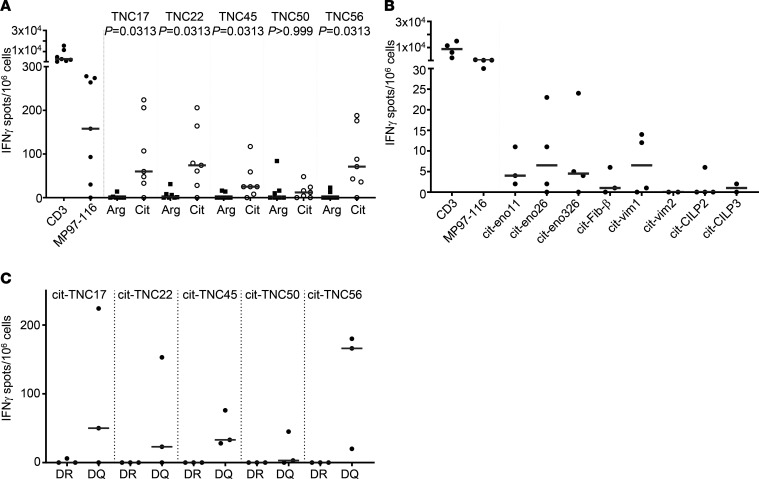
Synovial fluid mononuclear cells from patients with RA secrete IFN-γ in response to cit-TNC peptides. Synovial fluid mononuclear cells (SFMC) from patients with RA (*n* = 7) were stimulated with cit-TNC peptides or their arginine (arg) counterparts or other known cit-epitopes — cit-enolase (cit-eno), cit-fibrinogen (cit-Fib), cit-vimentin (cit-vim), cit-cartilage intermediate layer protein (cit-CILP) — for 48 hours. Influenza peptide (MP97-116) and anti-CD3 were used as positive controls. IFN-γ was measured by a 3-color FluoroSpot. The number of spots was normalized to spots per million cells, and spots seen in the unstimulated wells were subtracted from counts in the stimulated wells prior to further analyses. (**A**) Each symbol represents an individual subject, and the horizontal line shows the median SFMC from patients with RA produced IFN-γ in response to cit-TNC17, cit-TNC22, cit-TNC45, cit-TNC50, and cit-TNC56 peptides. (**B**) The level of IFN-γ produced was an order of magnitude greater than that produced in SFMC in response to cit-eno, cit-vim, and cit-CILP. Note that experiments in **A** and **B** were partly conducted in parallel, with 4 of the 7 subjects in **A** also included in **B**. Furthermore, apart from cit-eno26 and cit-eno326, the peptides between samples varied. (**C**) HLA-DR blocking antibodies (DR), but not HLA-DQ blocking antibodies (DQ), completely abrogated cit-TNC induction of IFN-γ secretion by SFMC from RA patients (*n* = 3). *P* values were calculated using a Wilcoxon matched pairs signed ranked test.

Having established that cit-TNC–specific T cells are present in peripheral blood, we next sought to evaluate their presence at disease-relevant sites of inflammation. Synovial fluid mononuclear cells (SFMC) from HLA-DRB1*04:01 RA patients (*n* = 7) were stimulated with the 5 immunogenic cit-TNC peptides or their corresponding arg-TNC peptides, and cytokine secretion (IFN-γ/IL-17/IL-10) was then assessed using a FluoroSpot assay. Citrullinated peptides derived from α-enolase, fibrinogen, vimentin, and cartilage intermediate-layer protein (CILP) were included as reference epitopes, since these proteins were previously reported as RA autoantigens (9, 11, 30–32) (Supplemental Table 4). Cit-TNC17, cit-TNC22, cit-TNC45, and cit-TNC56 (but not cit-TNC50) elicited IFN-γ secretion by SFMC (Figure 3A). Even though arg-TNC peptides could promote peripheral blood T cell expansion, we rarely observed cytokine responses to the unmodified peptides in the synovial fluid samples, while the cit-TNC peptides displayed a robust IFN-γ output (Figure 3A). Strikingly, the observed responses toward cit-TNC was almost an order of magnitude greater than that seen for cit-enolase, cit-fibrinogen, cit-vimentin, and cit-CILP (Figure 3B) — in some cases approaching the level of response seen for the influenza peptide. As expected, the observed SFMC response to cit-TNC peptides was HLA-DR restricted, since HLA-DR blocking — but not HLA-DQ blocking — completely abrogated the induction of IFN-γ secretion (Figure 3C). The cit-TNC peptides also elicited modest IL-10 and IL-17 secretion by SFMC (including some IFN-γ and IL-10 double positives), although this appeared to be epitope and subject specific (Supplemental Figure 2).

### Cit-TNC17 and cit-TNC56 are recognized by antibodies in RA subjects.

**Figure 4:**
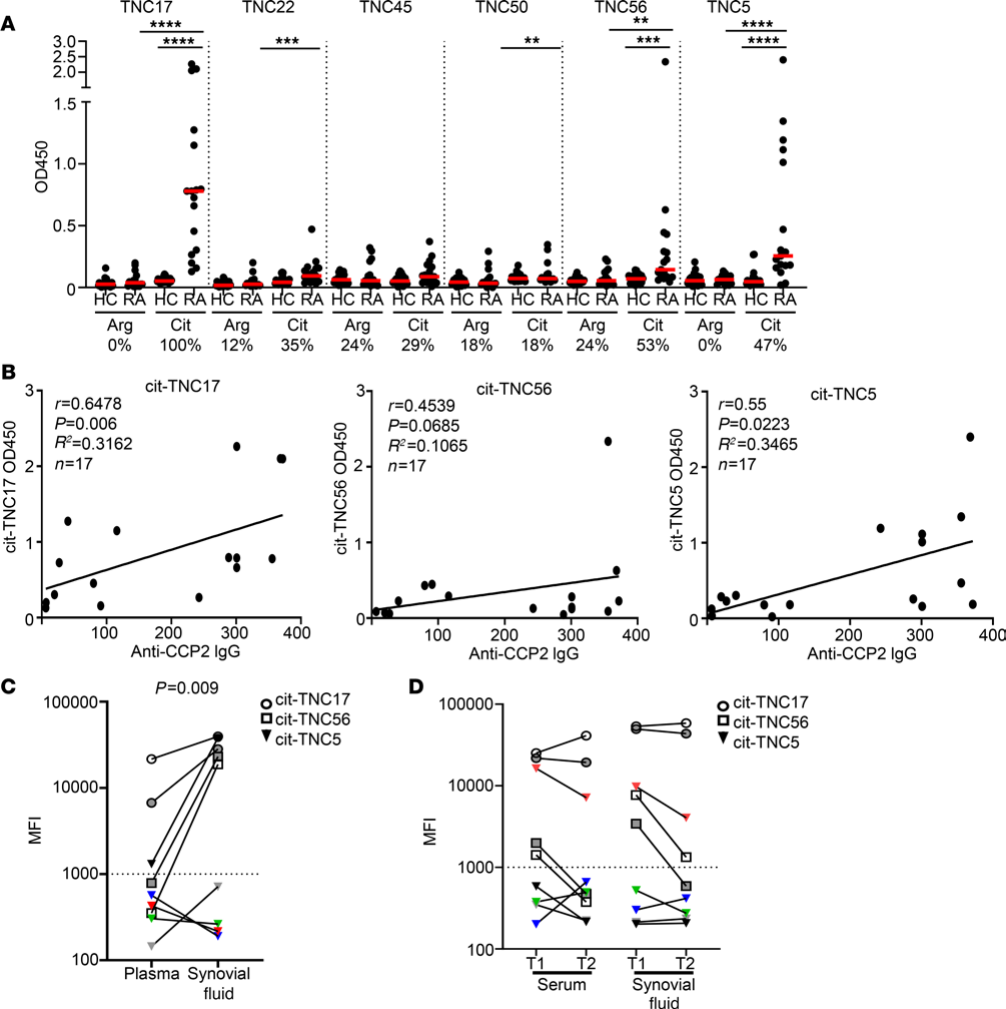
Cit-TNC17 and cit-TNC56 are citrullinated epitopes that are recognized by ACPA in patients with RA. Antibody responses to cit-TNC peptides or their arginine counterparts in serum from patients with RA and HC subjects were measured by ELISA. Numbers below *x* axis indicate percentage of subjects in each cohort that were seropositive. Each symbol represents an individual subject, and the horizontal line shows the median. (**A**) Antibodies to cit-TNC17, cit-TNC56 and cit-TNC5 were detected in 100%, 53%, and 47% of CCP^+^ RA subjects (*n* = 17), respectively, but not in HC subjects (*n* = 24), with little or no antibody response against the corresponding arg-TNC peptides. Each peptide was run on 1 plate, and each peptide pair (arg/cit) was run at the same day. *P* values were calculated using a Mann-Whitney *U* test. (**P < 0.01, ***P < 0.001, and ****P < 0.001). (**B**) CCP2 antibody levels correlated significantly with the antibody levels for both cit-TNC17 and cit-TNC5, but not cit-TNC56 (Spearman’s correlation coefficient, *P < 0.05). (**C**) Antibody reactivity to cit-TNC peptides was stronger in polyclonal anti-CCP pools from synovial fluid than plasma. Cutoff for positivity is 1000, and *P* values were calculated using a paired 2-tailed Student’s *t* test. (**D**) cit-TNC antibody reactivity over time in paired serum and synovial fluid samples from a single RA subject. T, time point. The different colors in **C** and **D** indicate the specific peptides tested in the microarray (see Supplemental Table 6).

Given the importance of ACPA as markers for the diagnosis of RA and prior observations that a citrullinated peptide from the C-terminal FBG domain of TNC is recognized by serum ACPA (18), we were curious about whether the peptides found to promote the T cell response to TNC were also antibody epitopes. To assess this, antibody responses to cit-TNC and arg-TNC peptides (plus the previously published cit-TNC5 peptide as a positive control) were measured in serum samples from HLA-DRB1*04:01 and cyclic citrullinated peptide^+^ (CCP^+^) RA (*n* = 17) and HLA-DRB1*04:01 matched HC (*n* = 24) subjects (Supplemental Table 2, Autoantibody Cohort 1, and Supplemental Table 5). By necessity, this autoantibody cohort had only partial overlap with the T cell cohort. Antibodies to the previously reported cit-TNC5 were present in 47% of the RA sera, consistent with the levels previously seen in 2 independent cohorts (22). In contrast, antibodies to cit-TNC17 were present in 100% of the RA sera, while antibodies to cit-TNC56, cit-TNC22, and cit-TNC45 were present in 53%, 35%, and 29% of the RA sera, respectively. Antibody titers were highest for TNC17, TNC5, and TNC56, while more modest reactivity was observed for TNC22 and TNC45 (Figure 4A). Notably, there was little or no antibody reactivity directed against the corresponding arg-TNC peptides (Figure 4A). Furthermore, antibody reactivity was virtually absent among the HC sera. These observations suggest that TNC is selectively recognized as a citrullinated antigen by antibodies present in the sera from RA patients that are essentially absent from HLA matched controls.

To further probe their potential significance, we correlated observed levels of cit-TNC–reactive antibodies with anti-CCP2 antibody levels (Figure 4B), observing a positive correlation in this cohort between anti-CCP2 and antibody levels for both cit-TNC17 (*r* = 0.6478, *P* = 0.006) and cit-TNC5 (*r* = 0.55, *P* = 0.0223), but that trend did not reach significance for cit-TNC56 (*r* = 0.4539, *P* = 0.0685). Since ACPA are thought to be directly involved in joint pathology, we next tested polyclonal anti-CCP pools purified from both plasma and synovial fluid samples for reactivity to an array of TNC peptides covering every arginine/citrulline residue within the protein (33). Among the peptides tested, we could verify reactivity to cit-TNC17, cit-TNC56, and cit-TNC5 in plasma and observed a strong positive signal in synovial fluid–derived anti-CCP pools (Figure 4C and Supplemental Table 6). The elevated levels of TNC reactivity in synovial fluid versus plasma were further replicated in a set of individually paired serum and synovial fluid samples taken 10 years apart, suggesting that cit-TNC antibodies persist over time (Figure 4D and Supplemental Table 6).

**Figure 5:**
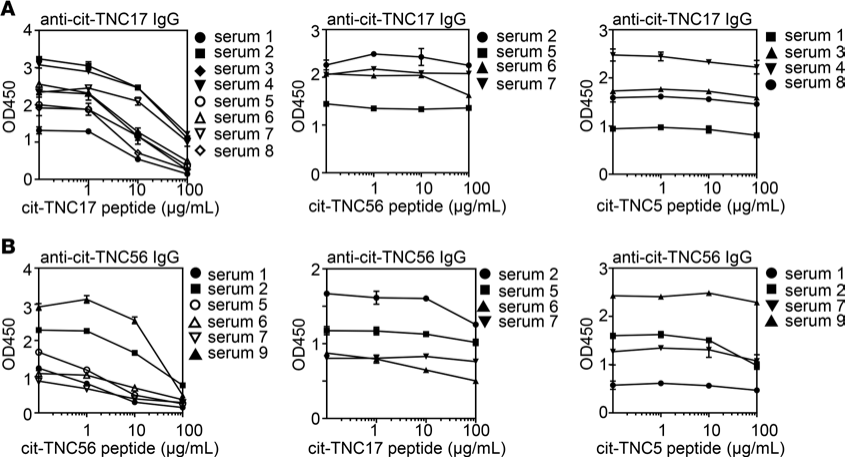
Anti–cit-TNC antibodies do not cross-react with each other. Sera that were double-reactive with peptides cit-TNC17 or cit-TNC56 and cit-TNC56, cit-TNC17, or cit-TNC5 were diluted 1:100, incubated with 1, 10, and 100 μg/mL of peptides for 2 hours and centrifuged at 10,000*g* for 10 minutes, and the supernatant then added to peptide-coated plates for analysis by ELISA. (**A**) No cross-reactivity between anti–cit-TNC17 and both anti–cit-TNC56 and anti–cit-TNC5. (**B**) Limited cross-reactivity between anti–cit-TNC56 and both anti–cit-TNC17 and anti–cit-TNC5. Each panel was run on a separate plate.

Since it has been recently shown that ACPA can cross-react against multiple peptides (especially those that contain a common citrulline-glycine motif; ref. 34), we examined the specificity and potential cross-reactivity between cit-TNC17– and cit-TNC56–reactive antibodies by performing inhibition experiments using sera from subjects that were double-reactive for cit-TNC17 and cit-TNC56 antibodies. Absorption by the homologous peptide was efficient for both cit-TNC17 and cit-TNC56 (Figure 5, left panels), while cross-reactivity was limited; only partial inhibition was observed in serum 2 (maximum inhibition: 24.8% for cit-TNC56 versus cit-TNC17) and serum 6 (maximum inhibition: 22% for cit-TNC17 versus cit-TNC56 and 43% for citTNC56 versus cit-TNC17) (Figure 5, middle panels). There was also no cross-reactivity between cit-TNC17 and cit-TNC5, and there was only limited cross-reactivity between cit-TNC56 and cit-TNC5 (maximum inhibition of serum 2: 38.7%) (Figure 5, right panels). Cumulatively, this minimal cross-reactivity suggests that distinct antibodies recognize these different cit-TNC peptides.

### Cit-TNC17 and cit-TNC56 antibodies are associated with clinical features of RA.

**Figure 6:**
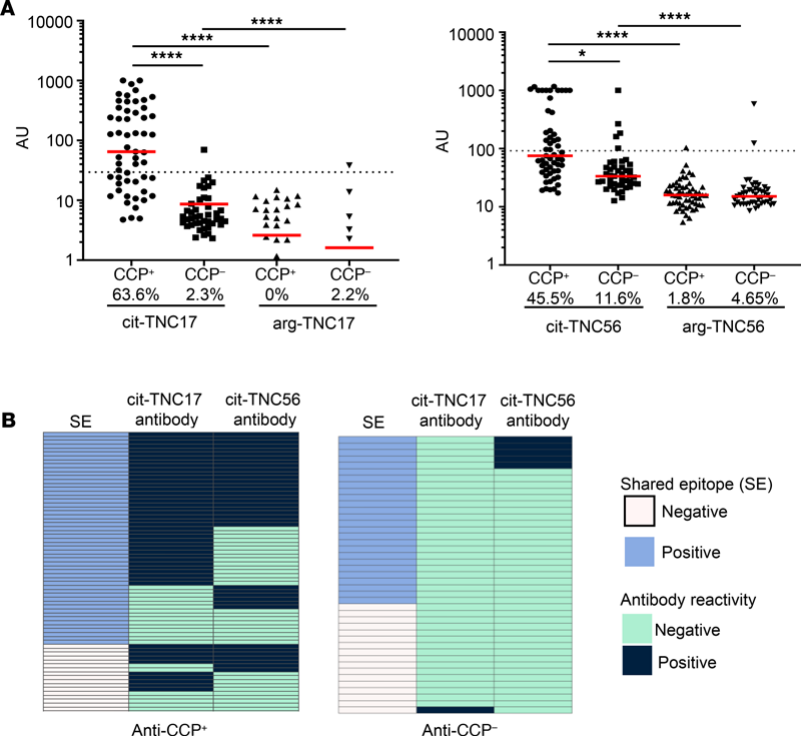
Cit-TNC antibodies are associated with CCP seropositivity and the shared epitope. Antibody responses to cit-TNC peptides or their arginine counterparts in serum from patients with RA and HC subjects were measured by ELISA. (**A**) cit-TNC17 and cit-TNC56 seropositivity was more prevalent in CCP^+^ RA subjects (*n* = 55) than CCP^–^ RA subjects (*n* = 43). Each symbol represents an individual subject, and the horizontal line shows the median. Dotted lines indicate cut-off for positivity. Numbers below *x* axis indicate percentage of each cohort that were seropositive. Left panel, cit-TNC17; cut off AU = 29.51. Right panel, cit-TNC56; cut off AU = 91.98. *P* values were calculated using a Kruskal Wallis test with Dunn’s multiple comparison test (*P < 0.05 and ****P < 0.001). (**B**) Heatmaps showing cit-TNC antibody seropositivity and its association with the HLA shared epitope (SE) in CCP^+^ RA subjects (*n* = 72; Autoantibody Cohorts 1 and 2 combined) and CCP^–^ RA patients (*n* = 43).

Given the prevalence of antibody reactivity toward cit-TNC17 and cit-TNC56 in subjects with RA, we investigated whether levels of these antibodies correlate with specific clinical features. To facilitate this analysis, we examined cit-TNC seropositivity in an independent cohort of RA subjects. This cohort included CCP^+^ RA (*n* = 55) and CCP^–^ RA (*n* = 43) subjects who were either positive or negative for the HLA DRB1*04:01 haplotype (Supplemental Table 2, Autoantibody Cohort 2). In this cohort, cit-TNC17 antibodies were present in 63.6% of CCP^+^ subjects but only 2.3% of CCP^–^ subjects, and cit-TNC56 antibodies were present in 45.5% of the CCP^+^ cohort and 11.6% of the CCP^–^ subjects (Figure 6A). The antibody reactivity for arg-TNC17 and arg-TNC56 peptides was below the threshold for positivity in almost all subjects (Figure 6A). Therefore, data from this patient cohort support preferred antibody reactivity toward cit-TNC and an association with CCP positivity.

We next combined the clinical and serologic data from Autoantibody Cohorts 1 and 2 to maximize our ability to assess relationships between cit-TNC17– and cit-TNC56–reactive antibodies with specific features of RA. In the combined cohort, 79% of the CCP^+^ subjects exhibited positive reactivity toward at least 1 of the cit-TNC peptides. Of these seropositive subjects, 28% were single positive for anti–cit-TNC17 antibodies, 11% were single-positive for anti–cit-TNC56 antibodies, and 40% were dual positive for both antibodies (Figure 6B). Furthermore, there was a positive association between cit-TNC seropositivity and the HLA SE (Figure 6B). This was further confirmed using a logistic regression model in which age and sex were used as covariates to calculate odds ratios (OR) (Table 3). In addition, cit-TNC17 seropositivity was strongly associated with both rheumatoid factor (OR = 14.2, FDR = 0.00002) and CCP seropositivity (OR = 92.98, FDR = 0.00009) (Table 3). To a lesser extent, cit-TNC56 seropositivity was also associated with rheumatoid factor (OR = 3.49, FDR = 0.04345) and CCP seropositivity (OR = 7.92, FDR = 0.0012) (Table 3). In addition, cit-TNC17 seropositivity was associated with smoking (current smoking OR = 6.5, FDR = 0.001) (Table 3) but not associated with disease activity and disease duration. Applying the model exclusively to SE^+^ RA subjects, the associations between cit-TNC17 seropositivity and rheumatoid factor, CCP seropositivity, and smoking remained strong and significant (Supplemental Table 7). The association of antibodies against cit-TNC17 and cit-TNC56 with the SE, smoking, and rheumatoid factor mirrors the characteristics described in ACPA^+^ subjects who go on to develop RA (3, 15, 35–41). Therefore, our results suggest that cit-TNC responsiveness is most pronounced in subjects with high-risk HLA genotypes and who have a history of smoking.

## Discussion

There is ample evidence to suggest that failure of both B cell and T cell tolerance contributes to the pathogenesis of seropositive RA. Indeed, in light of the observation of intimate interactions between T cells and B calls in the synovium (12) and the fact that antigen-specific B cells can be uniquely potent antigen-presenting cells, these are likely to be mutually reinforcing phenomena. ACPA and rheumatoid factor are diagnostic in RA, can precede disease by up to 10 years, and are thought to participate in joint pathology (2–4). ACPA have been shown to target multiple citrullinated antigens, with some exhibiting narrow or single-antigen specificity and others demonstrating substantial cross-reactivity toward shared consensus motifs (34). Notably, the number of ACPA specificities expands as individuals progress from pre-RA to develop inflammatory arthritis (4). CD4^+^ T cells are implicated through the strong genetic association of RA with HLA class II SE alleles, by their presence in the RA joint, and due to the affinity maturation and somatic mutation seen in RA autoantibodies indicating T cell–B cell collaboration (5–9, 11). This apparent collaboration, and observations that ACPA target multiple citrullinated antigens, have prompted the investigation of several citrullinated proteins as T cell targets in RA, including vimentin, α-enolase, CILP, fibrinogen, and aggrecan (42–47). The epitopes identified through these studies were typically recognized in the context of SE alleles, and in many cases, their immunogenicity depended on citrullination at key HLA-DR binding residues (in particular, binding pocket 4). Cumulatively, these studies have shown that T cells specific to citrullinated peptides are present in RA patients and exhibit an expanded memory phenotype.

Likewise, in this study, we explored the T cell response to TNC, an extracellular matrix protein with unique features that have been implicated in the development of RA, in the context of the RA risk allele HLA DRB1*04:01. Building on prior work that established cit-TNC5 as an important antibody epitope, we searched the entire TNC protein sequence to define immunogenic epitopes, identifying 5 potentially novel immunogenic T cell epitopes, 2 of which required citrulline modification for HLA binding and T cell recognition. We assessed the frequency of cit-TNC–specific T cells in peripheral blood through direct staining with HLA class II Tmrs, observing increased frequencies in RA subjects as compared with HC. Notably, a significant proportion of cit-TNC–reactive T cells in subjects with RA expressed CD38 (an activation marker on CD4^+^ memory T cells), suggesting expansion and recent activation. Furthermore, although some caution is advisable when comparing results obtained from different cohorts of patients, the observed frequency of cit-TNC–reactive T cells was substantially higher than those recently reported for other citrullinated antigens (e.g., memory frequencies of 2-6 cells per million for cit-aggrecan; ref. 10). T cells that recognized cit-TNC were also present among synovial fluid–derived T cells, exhibiting cytokine responses that were substantially more robust than established citrullinated epitopes. These findings suggest that cit-TNC is an important T cell target in RA.

Among the T cell epitopes identified in our study, 2 (TNC17 and TNC56) were also found to be antibody epitopes that were commonly recognized by serum antibodies from subjects with RA. These 2 epitopes (cit-TNC17 and cit-TNC56) were unique in that citrullination was required for peptide binding and immunogenicity. The relevance of these peptides as antibody targets was first demonstrated in a cohort of 17 HLA-DRB1*04:01^+^ and CCP^+^ RA patients. In those subjects, cit-TNC17–reactive antibodies were universally present, whereas cit-TNC56–reactive antibodies were present in just over half of the subjects (essentially the same proportion as the observed antibody reactivity against the previously published cit-TNC5 epitope). However, cit-TNC17– and cit-TNC56–reactive antibodies remained prevalent (64% and 43%, respectively) among CCP^+^ subjects in a larger cohort of 55 RA patients with more diverse HLA genotypes. Antibody recognition of these peptides was rare in CCP^–^ subjects, essentially absent in controls, and required citrullination. This requirement for citrullination of these peptides for both T cell and antibody recognition, along with the relatively high T cell frequencies observed, opens up the possibility of linked T cell and antibody responses that synergize in the setting of inflammation and tissue injury (known to promote protein citrullination by peptidyl arginine deiminase enzymes) to elicit uniquely robust responses. Naturally, these observations obtained through the use of citrullinated peptides should be interpreted with caution, given that relatively short peptides are not typically the natural targets of antibodies in vivo. However, although it could be argued that experiments using citrullinated WT recombinant TNC and recombinant TNC with mutations at key arginine residues are necessary to rigorously prove that these citrullinated sites are specifically targeted by antibodies in RA, our current observations are suggestive and informative.

Important insights that can be drawn from our study are the similarities and differences observed between peripheral blood and synovial samples. With the exception of cit-TNC50, each of the cit-TNC epitopes defined in peripheral blood drove strong cytokine responses in synovial fluid. However, although arg-TNC22–, arg-TNC45–, and arg-TNC50–specific T cells could be detected in peripheral blood, synovial T cell reactivity was exclusively observed in response to cit-TNC peptides. Further study would be needed to draw firm conclusions, but given that some studies suggest an enriched T cell repertoire in synovial fluid (48, 49), citrulline-reactive TCRs may be selectively retained within inflamed joints. Notably, although cit-TNC–specific T cells exhibited a memory phenotype in peripheral blood and synovial fluid, T cells with different phenotypes may home to different sites or be critical at different stages of disease. Specifically, in peripheral blood, cit-TNC–specific CD4^+^ memory T cells in RA subjects predominantly exhibited Th2-associated (CCR4^+^CCR6^–^CXCR3^–^) and Th17 (CCR4^+^CCR6^+^CXCR3^–^) surface phenotypes but very low proportions of Th1 (CXCR3^+^CCR4^–^CCR6^–^) cells. These 2 phenotypes could be interpreted to have opposing effects, given that Type 2 responses have been associated with remission (50), whereas IL-17 is thought to promote disease (51). Regrettably, our FluoroSpot assays were not designed to detect Th2 cytokines, with the exception of IL-10, which was only seen in a limited number of samples, whereas cit-TNC peptides did elicit robust IFN-γ responses from SFMC. Furthermore, prior studies have reported that the vast majority of T cells in synovial fluid are CXCR3^+^ (52). Therefore, it could be argued that cit-TNC–specific CXCR3^+^ T cells are enriched within inflamed joints and selectively reduced in the periphery. It is notable that antibody reactivity to both cit-TNC17 and cit-TNC56 was observed in RA serum and in polyclonal anti-CCP pools purified from both plasma and synovial fluid samples (including matched samples). Based on the consistently higher levels of reactivity seen for synovial fluid samples, it could be asserted that some B cells present within synovial infiltrates (53) produce cit-TNC–reactive antibodies.

In addition to documenting cit-TNC reactivity in general, our study sought to draw conclusions about the clinical significance of such responses. The observed associations between cit-TNC reactivity and CCP, RF, the SE, and smoking history are notable. In some sense, the positive relationship between cit-TNC–reactive antibodies and anti-CCP positivity is expected, given that polyclonal anti-CCP pools reacted to cit-TNC17, cit-TNC56, and cit-TNC5. Indeed, one confounding factor for cit-TNC17 in particular is the presence of a highly conserved citrulline-glycine “shared motif” within this peptide sequence that has been shown be recognized by highly multireactive ACPA (7, 34), such that these antibodies cannot be strictly defined as being cit-TNC specific. Therefore, it could be argued that an unknown proportion of cit-TNC17–reactive antibodies are multireactive ACPA with no discernible primary specificity. However, the cit-TNC56 peptide lacks the citrulline-glycine motif and is less widely recognized, but it still exhibits parallel positive relationships with CCP, RF, and the SE. This increases our confidence in the idea that cit-TNC responsiveness is most pronounced in subjects with high-risk HLA genotypes and who are RF^+^ and CCP^+^ — factors that have already been associated with erosive disease (2, 40, 54).

In summary, we have utilized complementary T cell and antibody measures to investigate cit-TNC as a relevant self-antigen in RA. We identified 5 immunogenic cit-TNC peptides that elicited T cell responses in the peripheral blood and synovial fluid of RA subjects, 2 of which were also recognized by ACPA from serum and synovial fluid. Cit-TNC responses were positively correlated with RF, CCP, and the SE, thereby linking these responses with a subgroup of patients known to have higher risk of severe joint destruction. It is clear that, for multiple citrullinated RA autoantigens, CD4^+^ T cell responses and corresponding ACPA responses to the same antigen have been documented. However, for cit-TNC in particular, we observe that the exact same cit-TNC peptides are recognized by both CD4^+^ T cells and B cells. We conclude that such shared recognition, coupled with the capacity for enhanced presentation by B cells where the BCR is specific for a peptide that then reinforces antigen-specific CD4^+^ T cell help to that same peptide, creates a unique scenario in which T cell–B cell cooperation in joints can amplify autoimmunity and promote the development and progression of RA.

## Methods

### Human subjects.

All RA subjects met the 2010 American College of Rheumatology (ACR)/European League Against Rheumatism (EULAR) 2010 Rheumatoid Arthritis Classification Criteria and had at least 1 HLA-DRB1*04:01 allele, and ACPA positivity was determined based on clinical testing for CCP. All HC subjects had no history of autoimmune disease themselves or among their first-degree relatives, had at least 1 HLA-DRB1*04:01 allele, and were ACPA^–^. The characteristics of each of the RA and HC cohorts are summarized in Supplemental Table 2. PBMC and serum samples from both RA and HC subjects were from the Benaroya Research Institute Immune-Mediated Disease Registry and Repository. Synovial fluid samples were obtained from patients with RA undergoing arthrocentesis as part of clinical treatment at the Karolinska University Hospital. For the study testing polyclonal anti-CCP pools purified from both plasma/serum and synovial fluid samples for reactivity to an array of TNC peptides, the plasma and serum samples were obtained from RA patients attending the Karolinska University Hospital as previously described (7, 33).

### Isolation and cryopreservation of PBMC and SFMC.

PBMC and SFMC were isolated from heparinized blood and synovial fluid, respectively, by centrifugation (1400*g*, room temperature, 15 minutes) over Ficoll-Hypaque (Thermo Fisher Scientific) gradients and were frozen in liquid nitrogen in 10% DMSO (MilliporeSigma) and 90% heat-inactivated FBS (Thermo Fisher Scientific). Cryopreserved PBMC were thawed in a 37°C water bath and prepared in drip-wise addition of RPMI-1640 (Thermo Fisher Scientific) media supplemented with 10% FBS and 0.001% benzonase nuclease (Sigma-Aldrich), with a final suspension in complete medium (RPMI-1640 supplemented with 2 mM L-glutamine [Thermo Fisher Scientific], 100 U/mL penicillin [Thermo Fisher Scientific], 100 μg/mL streptomycin [Thermo Fisher Scientific], 10 mM HEPES [MilliporeSigma]) supplemented with 10% commercial human pooled serum (HPS; Access Biologicals LLC). SFMC were thawed in a 37°C water bath and rapidly resuspended in complete medium with ≥ 5 U/mL benzonase nuclease (Sigma-Aldrich) and 10 % FBS. Before in vitro stimulation, the cells were resuspended in complete medium with 10 % HPS (Sigma-Aldrich).

### Epitope prediction and peptide synthesis.

Putative cit-TNC epitopes were predicted using our scanning algorithm as previously described (9, 24). Briefly, motif scores were calculated by multiplying coefficients corresponding to each anchor residue for all possible core 9-mers within the protein that included an internal or flanking arginine or citrulline residue. A total of 64 peptides with motif scores of 0.1 or higher were synthesized and purified by the manufacturer (Sigma-Aldrich). All peptides were dissolved in DMSO to a stock concentration of 20 mg/mL. For Tmr production and further studies, selected citrullinated peptides and their corresponding native peptides were resynthesized by Sigma-Aldrich, GenScript, or Pepceuticals.

### Peptide binding to HLA-DRB1*04:01.

To assess peptide binding to HLA-DRB1*04:01, increasing concentrations of each nonbiotinylated test peptide were incubated in competition with 0.01 μM biotinylated influenza HA306–318 (PKYVKQNTLKLAT) in wells coated with anti–HLA-DR antibody (clone L243, supplied by the BRI Tetramer Core) as previously described (55). Europium-conjugated streptavidin (PerkinElmer) was used to label residual biotinylated peptide bound to the HLA-DR protein and was quantified using a VICTOR2 multilabel time-resolved fluorometer (PerkinElmer). Binding curves were fitted by nonlinear regression with a sigmoidal dose response curve model using GraphPad Prism 7.0, and EC_50_ values were calculated as the peptide concentration needed to displace 50% of the reference peptide. Peptides selected for further study based on positive binding results were resynthesized at a higher purity by another manufacturer (GenScript or Pepceuticals).

### HLA class II Tmr production.

Recombinant HLA-DRB1*04:01 protein was produced by the BRI Tetramer Core as previously described (56). Soluble HLA-DRB1*04:01 monomer was purified from insect cell culture supernatants and biotinylated at a sequence-specific site using biotin ligase (Avidity) prior to dialysis into phosphate storage buffer. The biotinylated monomer was loaded with 0.2 mg/mL of peptide by incubating at 37°C for 72 hours in the presence of 2.5 mg/mL n-octyl β-D-glucopyranoside and 1 mM Pefabloc SC (Sigma-Aldrich). Peptide-loaded monomers were conjugated into Tmrs using fluorescently labeled streptavidin (Invitrogen) for 6–18 hours at room temperature at a molar ratio of 8:1.

### In vitro expansion cit-TNC–specific T cells and HLA class II Tmr staining.

Cryopreserved PBMCs from RA and healthy subjects were cultured at 5 × 10^6^ cells/well in a 48-well plate in RPMI-1640 + 10% HPS with 10 μg/mL of peptide. IL-2 (Novartis) was added at 325 IU/mL on day 6. On day 14, cells were stained for expression of Tmr-PE, CD25 APC (BD Biosciences), and CD4 FITC (BioLegend) (Supplemental Table 8) and then run on a FACSCanto. The data were analyzed by FlowJo software version 10.

### SFMC secretion of cytokines in response to cit-TNC peptides.

Cryopreserved SFMC from 7 patients were stimulated with cit-TNC17, cit-TNC 22, cit-TNC 45, cit-TNC 50, and cit-TNC56 peptides and their arginine counterparts. Peptide stimulation with citrullinated α-enolase, vimentin, CILP, and fibrinogen peptides was also made as a comparison for 4 of these 7 patients (*n* = 2 at the same time point as TNC peptides and *n* = 2 at a different time point and sample), plus 3 new patients in addition to the original 7 patients. Non-TNC peptides used in the assay were synthesized by GenScript and are described in Supplemental Table 4. One additional patient was only included in a TNC HLA-blocking experiment. A 3-color FluoroSpot assay was performed using the Human IFN-γ/IL-10/IL-17A FluoroSpot kit from Mabtech (catalog FSP-010703) for all TNC experiments, and the IFN-γ/IL-22/IL-17 kit was used for the additional experiments with other citrullinated peptides. Briefly, SFMC were thawed, and 250,000–850,000 cells were plated per well, in complete medium with 10 % human serum (Sigma-Aldrich), in a precoated FluoroSpot plate and stimulated with 20 μg/mL of the separate peptides for 48 hours at 37°C, 5% CO_2_. Influenza peptide (MP97-116; 20 μg/mL) and anti-CD3 (20 μg/mL) were used as positive control. HLA-DR (BioLegend; 10 μg/mL) and HLA-DQ (Beckman Coulter; 10 μg/mL) blocking antibodies were used in addition to the cit-TNC peptides for 3 patients. After incubation, the plate was washed in PBS and stained with antibodies according to the manufactures protocol. The plates were read using an ELISpot/FluoroSpot reader (iSpot Spectrum, AID), software version 7.0 (build 151117), with automated spot count. The number of spots was normalized to spots per million cells, and spots seen in the unstimulated wells were subtracted from spot count in the stimulated wells prior to further analyses. The Wilcoxon matched pairs signed rank test was used for the FluoroSpot assays, and *P* < 0.05 was considered significant.

### Ex vivo detection of influenza and cit-TNC–reactive T cells by HLA class II Tmrs.

Ex vivo Tmr staining and enrichment was accomplished using previously published protocols (10, 57). A total of 40 million–60 million PBMCs were thawed and rested overnight in a tissue culture incubator at 37°C and 5% CO_2_. PBMCs were equally divided into 2 FACS tubes for MP97-116 (influenza epitope) and cit-TNC Tmr tests in 200 μL of T cell culture medium following dasatinib (ChemieTek) treatment for 10 minutes at 37°C. PBMCs were then stained with 6 μL of each TNC-PE–, TNC-PE-CY5–, TNC-PE-CF–, or MP97-116-BV421–labeled Tmrs at room temperature for 90 minutes. Cells were washed and incubated with 40 μL anti-PE and 10 μL anti-Myc magnetic beads (Miltenyi Biotec) at 4°C for 20 minutes; they were washed again, and a 1/100 fraction was saved for antibody staining (“Pre”). The other fraction was passed through a MS magnetic column (Miltenyi Biotec). Bound PE-, PE-CY5–, PE-CF–, or BV421-labeled cells were flushed and collected. Both enriched (“Post”) and nonenriched (“Pre”) fractions were labeled with Sytox, CD4 V500, CD45RA AF700, or CD38 BUV395 (all from BD Bioscience) or with CXCR3 AF647, CCR4 BV605, CXCR5 PE-Cy7, CCR6 BV650, CCR7 APC-Cy7, CD14 FITC, or CD19 FITC (all from BioLegend) (Supplemental Table 8). Samples were run on a BD LSRII flow cytometer, and data were analyzed using FlowJo software version 10.

The frequency (F) of epitope-specific T cells per million CD4^+^ memory T cells was calculated as follows: F = (1,000,000 × Tmr^+^ events from enriched tube)/(100 × number of CD4^+^ memory T cells from the “Pre” fraction). Single T cells were gated CD4^+^/CD14^−^ CD19^−^sytox^−^, and a triple-negative population was gated for each of the 3 possible Tmr fluorophores. These served as the parent populations, from which 1 of the 4 Tmr^+^ T cell populations was then gated on and analyzed for surface receptor expression. T cell lineage was assigned on CD45RA^−^ memory cells as follows: Th1 (CXCR3^+^, CCR4^−^, and CCR6^−^); Th2 (CXCR3^−^, CCR4^+^, and CCR6^−^); Th17 (CXCR3^−^, CCR4^+^, and CCR6^+^); and Th1* (CXCR3^+^, CCR4^−^, and CCR6^+^) (25, 26).

### ELISAs for detection of antibodies.

ELISAs were used to detect antibodies against citrullinated peptides in sera from patients in Autoantibody Cohorts 1 and 2 (Supplemental Table 2). Peptides used in the assay were synthesized by either GenScript or Pepceutical and are described in Supplemental Table 5. The ELISAs were conducted as previously described (18, 26). In brief, 96-well Nunc Maxisorp plates were coated with 10 μg/mL peptide in PBS, blocked with 2% BSA, and incubated with sera diluted 1:100. Bound antibodies were detected with an HRP-conjugated anti–human IgG Fc monoclonal antibody (6043HRP, Stratech). A standard curve of positive sera was used to calculate relative antibody titers in arbitrary units (AU) for each sample. The CCP2 ELISA for Autoantibody Cohort 1 was performed as by the manufacturer’s instruction (FCCP600, Axis-Shield). Cross-reactivity to cit-TNC56, cit-TNC17, or cit-TNC5 was analyzed in human sera reactive to cit-TNC56 or cit-TNC17. Sera were diluted 1:100, incubated with 1, 10, and 100 μg/mL of peptides for 2 hours and centrifuged at 10,000*g* for 10 minutes at room temperature, and the supernatant was added to peptide-coated plates for analysis by ELISA as described above. Positivity was defined by the cut-off of the 98th percentile of HC samples from Autoantibody Cohort 1.

### Extracellular matrix peptide microarray.

The custom-made microarray was performed as previously described (7). In brief, 16 amino acid–long peptides that covered all arginine residues of 1610 extracellular matrix proteins and RA-related proteins were synthesized in situ (Roche NimbleGen). For TNC, there were a total of 217 peptides, including both native and citrullinated variants of each peptide synthesized. Samples tested for reactivity against TNC-derived peptides included synovial and plasma ACPA pools containing CCP-reactive antibodies from 26 and 38 RA patients, respectively (33, 34) Paired synovial fluid and serum samples from 1 RA patient taken at 2 time points 10 years apart were also screened. ACPA pools were run at a concentration of 15 μg/mL, whereas synovial fluid and plasma or serum samples were diluted 1/100 and tested for citrulline reactivity using a NimbleGen MS200 Scanner (Roche NimbleGen). A peptide signal intensity variation (spot size) of 25 pixels was used as a basis for calculating the median fluorescence intensities. The cutoff for positive signals was defined as 5× the fluorescence intensity of the 98th percentile of values for a set of monoclonal antibodies without citrulline reactivity. These were included in the same experiment and were tested at the same time.

### Statistics.

All statistical tests were performed using GraphPad Prism version 7.02. Tests that were used (as appropriate) included paired 2-tailed Student’s *t* tests, Wilcoxon matched pairs singed ranked test, Spearman’s correlations, Mann-Whitney *U* tests, and Kruskal Wallis test with Dunn’s multiple comparison test. *P* < 0.05 was considered significant. To determine the association of TNC seropositivity with clinical variables — including smoking, HLA-DRB1 SE, Routine Assessment of Patient Index Data 3 (RAPID3) disease activity scores, disease duration, and the protein tyrosine phosphatase nonreceptor type 22 gene (PTPN22) genetic variant C1858T SNP. OR with 95% Wald confidence intervals (95% CI) were calculated using logistic regression models including age and sex as covariates. *P* values were corrected for multiple testing with the Benjamini-Hochberg method.

### Study approval.

The study was approved by Benaroya Research Institute’s IRB (protocol no. IRB07109) and the Karolinska University Hospital’s IRB (protocol no. dnr 03-138). All samples were obtained under approved research protocols with informed consent.

## Author contributions

JS, KSM, VM, EAJ, and JHB conceptualized and designed the study. JHB and JS were responsible for subject selection and clinical data collection. JS performed the T cell studies. CR and DAL performed peptide binding assay. ST selected samples for and performed the synovial fluid studies. CG performed the analyses of the peptide array data. AS, AW, and LRM performed the autoantibody studies. VSM performed the statistical modeling. JS, EAJ, and JHB wrote the manuscript with assistance from all coauthors. JHB obtained funding and was responsible for the entire project.

## Supplementary Material

Supplemental data

## Figures and Tables

**Table 1:**
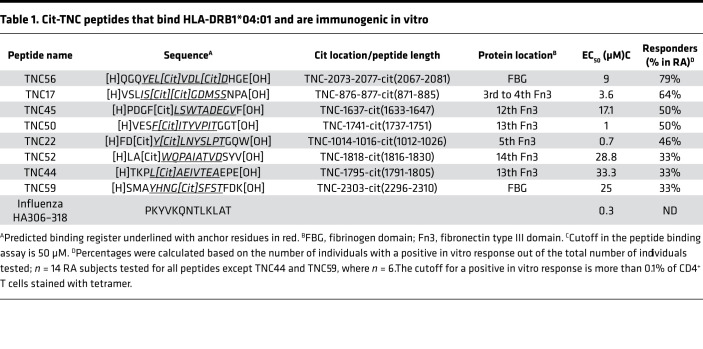
Cit-TNC peptides that bind HLA-DRB1*04:01 and are immunogenic in vitro

**Table 2:**
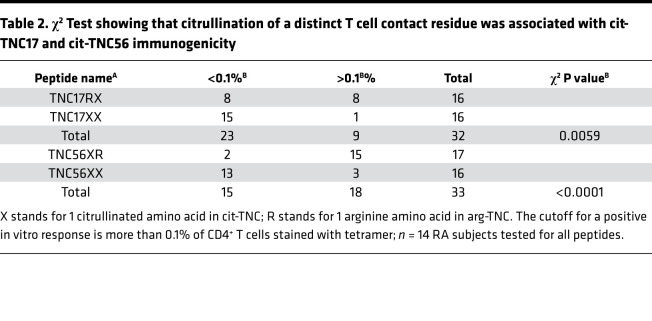
χ^2^ Test showing that citrullination of a distinct T cell contact residue was associated with cit-TNC17 and cit-TNC56 immunogenicity

**Table 3:**
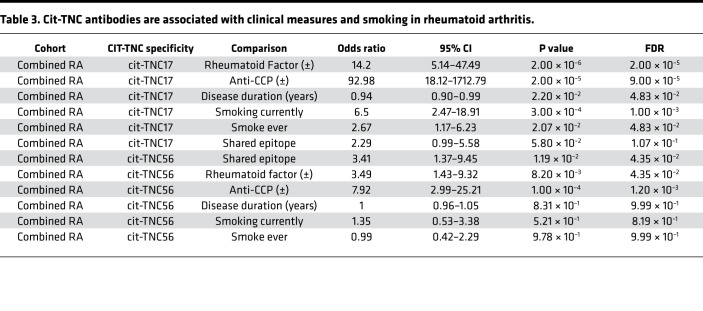
Cit-TNC antibodies are associated with clinical measures and smoking in rheumatoid arthritis.

## Version 1 01/28/2021

In-Press Preview

## Version 2 03/08/2021

Electronic publication
