# Supplementary material for: Shared recognition of citrullinated tenascin-C peptides by T and B cells in rheumatoid arthritis
Source: JCI Insight. 2021 Mar 8;6(5):e145217. doi: 10.1172/jci.insight.145217 (PMC8021118; doi:10.1172/jci.insight.145217)
Supplement: Supplemental data [file jciinsight-6-145217-s244.pdf]

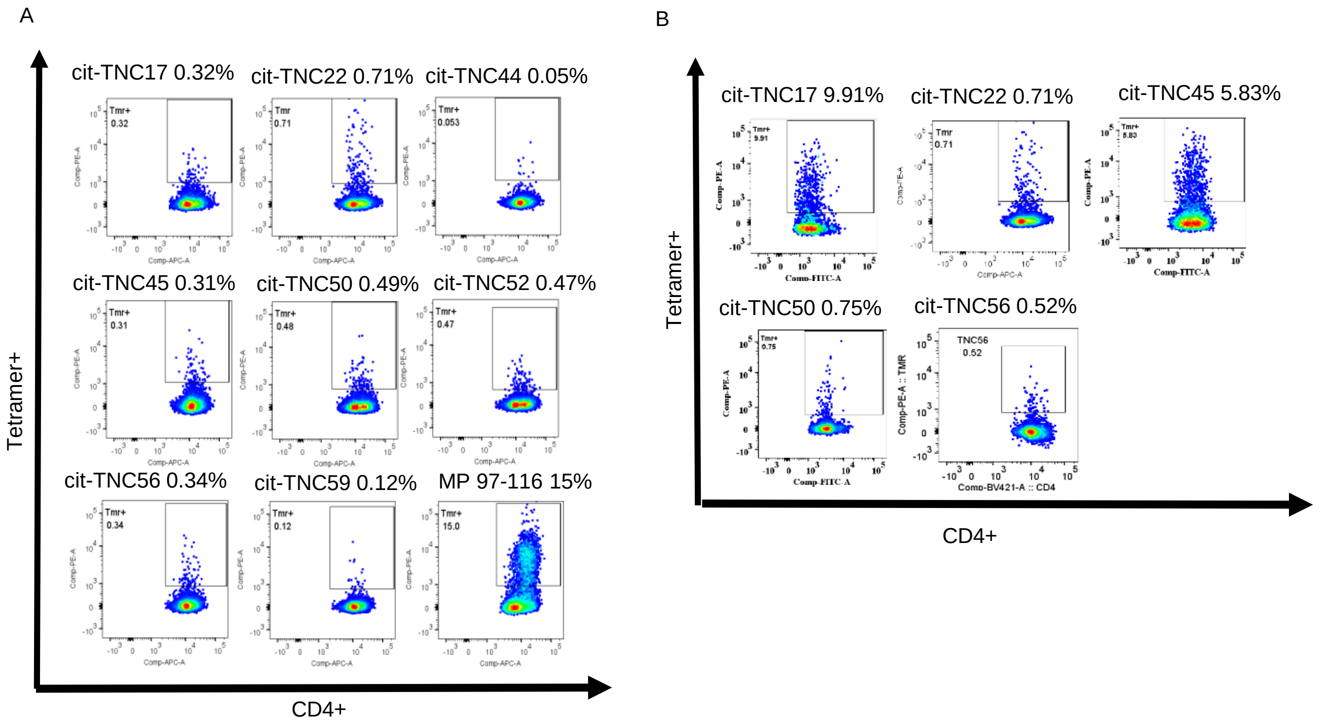

Supplemental Figure 1. cit-TNC peptides exhibit *in vitro* immunogenicity. Cit-TNC peptides were evaluated for immunogenicity by stimulating PBMC from subjects with HLA-DRB1*04:01 haplotypes for 14 days with peptide and then staining with the corresponding HLA class II tetramers. (**A**) Representative plots showing populations of tetramer+ T cells following expansion in response to eight cit-TNC peptides or the positive control influenza peptide MP 97-116 in a single RA subject. *In vitro* cultures with greater than 0.1% tetramer positive CD4+ T cells were considered positive. (**B**) Best representative positive staining for each of the five cit-TNC peptides that were found to be immunogenic from 5 different RA subjects. Note the subject shown in (A) had the best representative plot for cit-TNC22 so this plot is the same in both A and B.

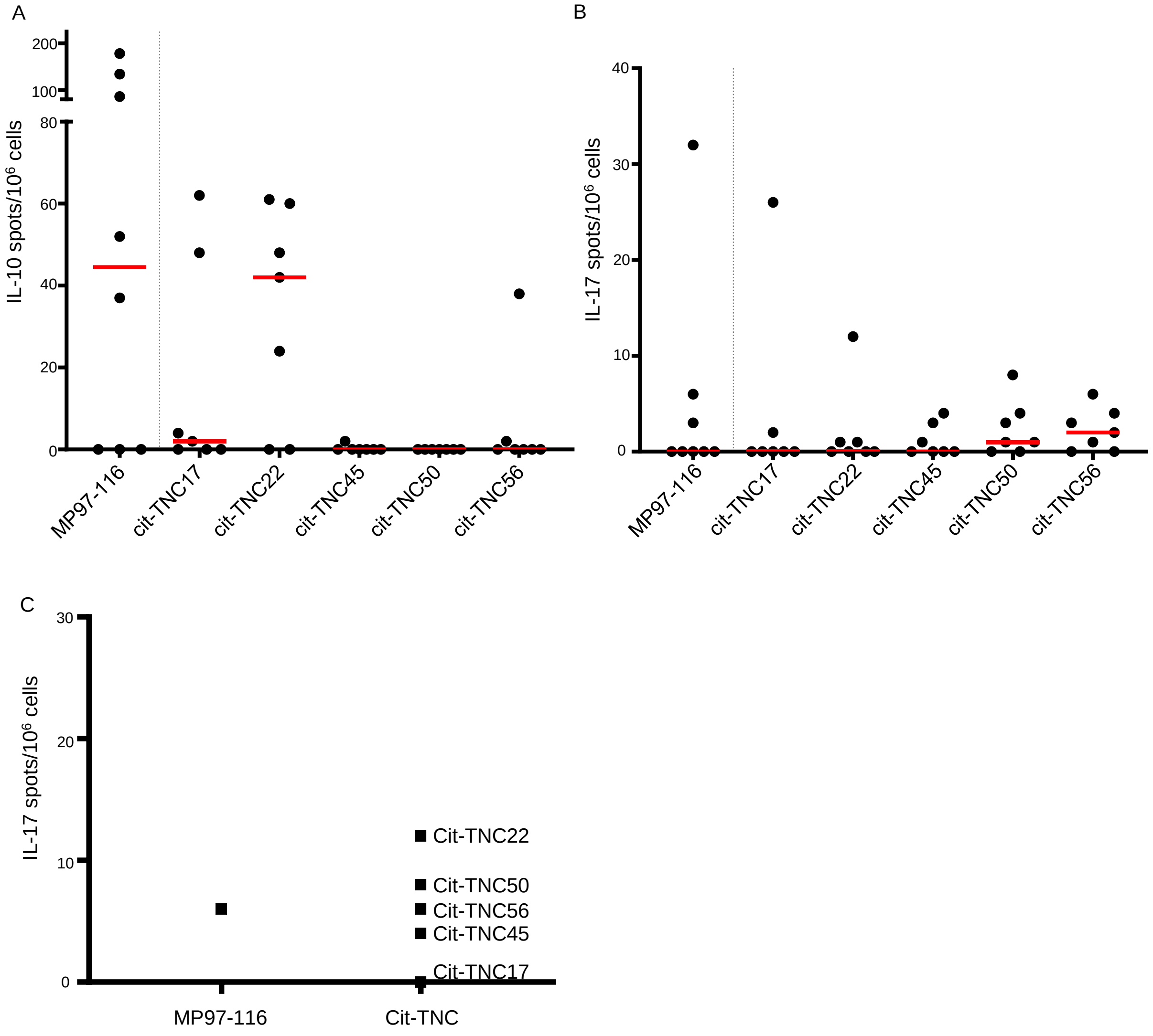

Supplemental Figure 2. Synovial fluid mononuclear cells from patients with RA also secrete IL-10 and IL-17 in response to cit-TNC peptides. Synovial fluid mononuclear cells (SFMC) from patients with RA (n=7) were stimulated with cit-TNC peptides or their arginine counterparts for 48 hours. Influenza peptide (MP54) and CD3 (not shown) were used as positive controls. IL-10 and IL-17 production was measured by a three-color fluorospot. Number of spots were normalized to spots per million cells and spots seen in the unstimulated wells were subtracted from count in the stimulated wells prior to further analyses. Each symbol represents an individual subject, and the horizontal line shows the median. **(A)** SFMC from patients with RA (n=7) produced IL-10 in response to cit-TNC17, and cit-TNC22. **(B)** SFMC from patients with RA (n=7) produced IL-17 in response to cit-TNC50, and cit-TNC56. **(C)** A predominant IL-17 response in a single RA subject with 4/5 of the cit-TNC peptides inducing SFMC to secrete IL-17 with no induction of IFN-γ or IL-10 secretion by any of the cit-TNC peptides (data not shown).

Supplemental Table 1. Effect of citrullination on the ability of the TNC peptide to bind HLA-DRB1*0401 and to elicit an immune response i*n vitro*

Peptide	Sequence[A]	EC50 (μM)[B]	Immunogenicity
TNC-17XX	[H]VSL*IS* ***[Cit][Cit]*** *GDMSS* NPA[OH]	3.6	Yes
TNC-17RX	[H]VSL*IS* ***R[Cit]*** *GDMSS* NPA[OH]	4.7	No
TNC-17XR	[H]VSL*IS* ***[Cit]R*** *GDMSS* NPA[OH]	>50	ND
TNC-17RR	[H]VSL*IS* ***RR*** *GDMSS* NPA[OH]	>50	ND
TNC-22XX	[H]FD**[Cit]***Y* ***[Cit]*** *LNYSLPT* GQW[OH]	0.7	Yes
TNC-22RX	[H]FD**R***Y* ***[Cit]*** *LNYSLPT* GQW[OH]	0.9	Yes
TNC-22XR	[H]FD**[Cit]***Y* ***R*** *LNYSLPT* GQW[OH]	0.6	Yes
TNC-22RR	[H]FD**R***Y* ***R*** *LNYSLPT* GQW[OH]	1	Yes
TNC-45X	[H]PDGF**[Cit]***LSWTADEGV* F[OH]	17.1	Yes
TNC-45R	[H]PDGF**R***LSWTADEGV* F[OH]	5.7	Yes
TNC-50X	[H]VES*F* ***[Cit]*** *ITYVPIT* GGT[OH]	1	Yes
TNC-50R	[H]VES*F* ***R*** *ITYVPIT* GGT[OH]	1	Yes
TNC-56XX	[H]QGQ*YEL* ***[Cit]*** *VDL* ***[Cit]*** *D* HGE[OH]	9	Yes
TNC-56RX	[H]QGQ*YEL* ***R*** *VDL* ***[Cit]*** *D* HGE[OH]	>50	ND
TNC-56XR	[H]QGQ*YEL* ***[Cit]*** *VDL* ***R*** *D* HGE[OH]	8.7	No
TNC-56RR	[H]QGQ*YEL* ***R*** *VDL* ***R*** *D* HGE[OH]	>50	ND

A. Predicted binding register underlined with anchor residues in red
B. Cutoff in the peptide binding assay is 50μM

Supplemental Table 2. Characteristics of cohorts

**T CELL COHORT**	**RA (n=9)**	**HC (n=7)**
Age at Draw (Median, Range: Min-Max)	53 yrs. (32-87 yrs.)	46 yrs. (28-69 yrs.)
Male/Female (number of subjects)	2/7	4/3
Disease Duration (Mean±SD)	3.98±2.23 yrs.	
Ever Smoker/Never Smoker/Unknown (number of subjects)	4/5/0	
**AUTOANTIBODY COHORT 1**	**RA (n=17)**	**HC (n=24)**
Age at Draw (Median, Range: Min-Max)	52 yrs. (31-87 yrs.)	40.5 yrs. (23-69 yrs.)
Male/Female (number of subjects)	5/12	8/16
Disease Duration (Mean±SD)	3.23±1.91 yrs.	
Ever Smoker/Never Smoker/Unknown (number of subjects)	6/11/0	
**AUTOANTIBODY COHORT 2**	**CCP^{pos} RA (n=55)**	**CCP^{neg} RA (n=43)**
Age at Draw (Median, Range: Min-Max)	56 yrs. (22-87 yrs.)	56 yrs. (29-89 yrs.)
Male/Female (number of subjects)	14/41	9/34
Disease Duration (Mean±SD)	10.62±10.26 yrs.	10.36±9.42 yrs.
Ever Smoker/Never Smoker/Unknown (number of subjects)	26/28/1	17/21/5
**SYNOVIAL FLUID COHORT**	**$ACPA^{pos}$ RA (n=11)**	
Age at Draw (Median, Range: Min-Max)	54 yrs. (39-65 yrs.)	
Male/Female (number of subjects)	3/8	

Supplemental Table 3. cit-TNC-specific memory CD4+ T cells detected directly *ex vivo* are more frequent in the peripheral blood from RA subjects than healthy control subjects

	Healthy Control Subjects (n=7)		RA Subjects (n=9)	
**Peptide tested**	**Percentages[A]**	**Frequencies[B]**	**Percentages**	**Frequencies**
Pooled TNC	ND	15.9± 5.4	ND	108.3± 44.6 (*P-value=0.0048*)
cit-TNC-17	71% (5/7)	2.1± 1.0	78% (7/9)	8.1±3.7
cit-TNC-22	71% (5/7)	3.0±1.2	56% (5/9)	3.8±1.9
cit-TNC-45	71% (5/7)	2.6±1.0	100% (9/9)	22.6±6.4 (*P-value=0.0012*)
cit-TNC-50	71% (5/7)	1.5±0.6	100% (9/9)	9.6±3.1 (*P-value=0.0045*)
cit-TNC-56	57% (4/7)	1.2±0.5	100% (9/9)	47.4±26.0 (*P-value<0.0001*)
MP97-116	100% (7/7)	126.3±37.4	100% (9/9)	91.7±19.8

A. Percentages calculated based on the number of subjects with a detectable *ex vivo* response per total numbers of subjects tested.
B. Mean±SEM/million memory CD4+ T cells

Supplemental Table 4. Sequences of peptides from a-enolase, CILP, fibrinogen and vimentin used to stimulate synovial fluid mononuclear cells

Peptide Name	Protein source	Peptide Location	Sequence
cit-eno-11	α-enolase	cit11-25	IFDS[Cit]GNPTVEVDLF
cit-eno-26	α-enolase	cit26-40	TSKGLF[Cit]AAVPSGAS
cit-eno-326	α-enolase	cit326-340	K[Cit]IAKAVNEKSCNCL
arg-eno-26	α-enolase	arg-eno 26-40	TSKGLFRAAVPSGAS
arg-eno-326	α-enolase	arg-eno 326-340	KRIAKAVNEKSCNCL
cit-CILP2	CILP	cit-CILP297-311	ATIKAEFV[Cit]AETPYM
cit-CILP3	CILP	cit-CILP982-979	GKLYGI[Cit]DV[Cit]STRDR
Cit-Fib b	Fibrinogen	cit-fib b 69-80	GY[Cit]A[Cit]PAKAAAT
Cit-Vim-1	Vimentin	cit-vim59-78	GVYAT[Cit]SSAV[Cit]L[Cit]SSVPGVR
Cit-Vim-2	Vimentin	cit-vim418-431	FSSLNL[Cit]ETNLDSL

Supplemental Table 5. Sequences of peptides used for detection of anti-citrulline protein antibodies

Peptide Name	Peptide Sequence
cit-TNC5 (Ref. 21)	CEHSIQFAEMKL[Cit]PSNF[Cit]NLEG[Cit][Cit]KRC
arg-TNC5 (Ref. 21)	CEHSIQFAEMKLRPSNFRNLEGRRKRC
cit-TNC17	CEYEVSLIS[Cit][Cit]GDMSSNPAC
arg-TNC17	CEYEVSLISRRGDMSSNPAC
cit-TNC22	CTPLAKFD[Cit]Y[Cit]LNYSLPTGC
arg-TNC22	CTPLAKFDRYRLNYSLPTGC
cit-TNC45	CVSDATPDGF[Cit]LSWTADEGC
arg-TNC45	CVSDATPDGFRLSWTADEGC
cit-TNC50	CPTAQVESF[Cit]ITYVPITGGC
arg-TNC50	CPTAQVESFRITYVPITGGC
cit-TNC56	CQGQYEL[Cit]VDL[Cit]DHGETAFC
arg-TNC56	CQGQYELRVDLRDHGETAFC

Supplemental Table 6. Sequences of peptides tested in microarray used in Figures 4C and 4D

TNC17 XR: YEVSLIS[Cit]RGDMSSNP
TNC17 RX: YEVSLISR[Cit]GDMSSNP
TNC56 XR: QYEL[Cit]VDLRDHGETAF
TNC56 RX: QYELRVDL[Cit]DHGETAF
TNC5 cit2187: IQFAEMKL[Cit]PSNFRNL
TNC5 cit2192: MKLRPSNF[Cit]NLEGRRK
TNC5 cit2197: LRPSNFRNLEG[Cit]RKRA
TNC5 cit2198: LRPSNFRNLEGR[Cit]KRA
TNC5 cit2199: LRPSNFRNLEGRRK[Cit]A

Supplemental Table 7. cit-TNC17 antibodies are associated with clinical measures and smoking in a cohort of RA subjects that are positive for the HLA shared epitope

Cohort	CIT-TNC specificity	Comparison	Odds Ratio	95% Confidence Intervals	P Value	FDR
Shared Epitope	cit-TNC17	Rheumatoid Factor (+/-)	12.91	3.90 - 53.69	1.00E-04	1.10E-03
Shared Epitope	cit-TNC17	Anti-CCP (+/-)	5.1	1.76 - 17.3	4.60E-03	4.56E-02
Shared Epitope	cit-TNC17	Disease Duration (years)	0.93	0.86-0.98	1.38E-02	4.41E-02
Shared Epitope	cit-TNC17	Smoking Currently	12.18	2.91 - 84.77	2.40E-03	1.18E-02
Shared Epitope	cit-TNC17	Smoke Ever	3.52	1.27 - 10.34	1.76E-02	4.41E-02
